# Homogalacturonan Pectins Tuned as an Effect of Susceptible rbohD, Col-0-Reactions, and Resistance rbohF-, rbohD/F-Reactions to TuMV

**DOI:** 10.3390/ijms25105256

**Published:** 2024-05-11

**Authors:** Katarzyna Otulak-Kozieł, Edmund Kozieł, Krzysztof Treder, Piotr Rusin

**Affiliations:** 1 Department of Botany, Institute of Biology, Warsaw University of Life Sciences—SGGW, Nowoursynowska Street 159, 02-776 Warsaw, Poland; 2 Plant Breeding and Acclimatization Institute—National Research Institute in Radzików, Bonin Division, Department of Potato Protection and Seed Science at Bonin, Bonin Str. 3, 76-009 Bonin, Poland; k.treder@ihar.edu.pl

**Keywords:** cell wall changes, pectin, plant-virus interactions, ultrastructure

## Abstract

The plant cell wall is an actively reorganized network during plant growth and triggered immunity in response to biotic stress. While the molecular mechanisms managing perception, recognition, and signal transduction in response to pathogens are well studied in the context of damaging intruders, the current understanding of plant cell wall rebuilding and active defense strategies in response to plant virus infections remains poorly characterized. Pectins can act as major elements of the primary cell wall and are dynamic compounds in response to pathogens. Homogalacturonans (HGs), a main component of pectins, have been postulated as defensive molecules in plant–pathogen interactions and linked to resistance responses. This research focused on examining the regulation of selected pectin metabolism components in susceptible (*rbohD*-, Col-0-TuMV) and resistance (*rbohF*-, *rbohD/F*–TuMV) reactions. Regardless of the interaction type, ultrastructural results indicated dynamic cell wall rebuilding. In the susceptible reaction promoted by RbohF, there was upregulation of *AtPME3* (pectin methylesterase) but not *AtPME17*, confirmed by induction of PME3 protein deposition. Moreover, the highest PME activity along with a decrease in cell wall methylesters compared to resistance interactions in *rbohD*–TuMV were noticed. Consequently, the susceptible reaction of *rbohD* and Col-0 to TuMV was characterized by a significant domination of low/non-methylesterificated HGs. In contrast, cell wall changes during the resistance response of *rbohF* and *rbohD/F* to TuMV were associated with dynamic induction of *AtPMEI2*, *AtPMEI3*, *AtGAUT1*, and *AtGAUT7* genes, confirmed by significant induction of PMEI2, PMEI3, and GAUT1 protein deposition. In both resistance reactions, a dynamic decrease in PME activity was documented, which was most intense in *rbohD/F*–TuMV. This decrease was accompanied by an increase in cell wall methylesters, indicating that the domination of highly methylesterificated HGs was associated with cell wall rebuilding in *rbohF* and *rbohD/F* defense responses to TuMV. These findings suggest that selected PME with PMEI enzymes have a diverse impact on the demethylesterification of HGs and metabolism as a result of *rboh*–TuMV interactions, and are important factors in regulating cell wall changes depending on the type of interaction, especially in resistance responses. Therefore, PMEI2 and PMEI3 could potentially be important signaling resistance factors in the *rboh*–TuMV pathosystem.

## 1. Introduction

The plant cell wall is a highly dynamic and complex structure that undergoes active changes during development, providing support for cell-to-cell communication and responding to biotic and abiotic stresses [1]. Plant cell wall heterogenous structures are composed of interacting proteins, polysaccharides, phenolic compounds, and water content, and play a crucial role in determining cell shape and directing growth. Additionally, the plant cell wall serves as a modulating system and signaling hub in plant immunity, particularly during biotic stress conditions [2,3]. Extensive research has been conducted on the molecular mechanisms underlying the structural and functional integrity of the cell wall, as well as the perception, recognition, and signal transduction pathways involved in triggered immunity through membrane receptors and effectors [4,5,6,7,8,9]. Bellicampi et al. [10] described the cell wall as a battleground where interactions between plant cells and pathogens can lead to either cell wall weakening or reinforcement. Fungi produce a variety of cell wall-degrading enzymes [11], while soft rot bacteria synthesize important wall-degrading enzymes that contribute to their virulence [12]. Parasitic nematodes and phytophagous insects also induce wall-degrading enzymes for their invasions [13]. Despite the progress in understanding the role and composition of the cell wall in response to damaging intruders, our knowledge of plant cell wall changes and active defense strategies in response to plant virus infections remains incomplete.

Recent research based on transcriptomic and proteomic analyses of plant responses to virus pathogens has shed light on the importance of the cell wall in defense responses to virus stress [14,15,16,17,18,19,20,21,22,23]. These studies indicate that genes and transcripts can be significantly differentially regulated during these interactions. Previous research has shown variable distributions of β-1,3-glucanase (PR-2), the catalytic subunit of cellulose synthase A4 (CesaA4), or dynamically changed xyloglucan/xylan metabolism with the participation of xyloglucan transferase XTH-Xet5 in the potato virus y (PVY^NTN^)–potato pathosystem [24,25]. Furthermore, it has been observed that cell wall components’ spatial and temporal depositions are strictly associated with the type of reaction to the virus. Studies have also postulated that *Solanum tuberosum* expansin StEXPa and hydroxyproline-rich glycoprotein (HRGP) extensins are differentially regulated and intracellularly distributed depending on compatible (susceptible) or incompatible (hypersensitive response) interactions between PVY^NTN^ and potato plants [26]. These observations confirmed that the susceptible reaction to PVY^NTN^ was associated with potato cell wall loosening, whereas, during the hypersensitive response, cell wall strengthening was noticed. Additionally, further analyses during the response of NADPH oxidase homologs D and F deficient mutants (*rbohD*, *rbohF*, and *rbohD/F*) to TuMV highlighted cell wall changes [27]. The data indicated that not only the symplast but also the apoplast was involved in the activation of the response to potyvirus. According to the observations, the apoplast was dynamically rebuilt during susceptible *rbohD*–TuMV and Col-0-TuMV interactions, as well as during the limiting virus content in *rbohF*–TuMV and *rbohD/F*–TuMV resistance reactions. It was suggested that RbohD could be involved in the resistance response to TuMV, whereas RbohF promotes susceptibility in that pathosystem. Finally, it was reported that glutathione participated in an active reaction to TuMV, confirming the assumption that, in *rboh* mutants, the apoplast is involved in the signaling defense response to TuMV stress [28]. Active changes in the apoplast, and especially alterations in the structure and composition of the cell wall, affected plant resistance to biotic stress [29].

Pectins in the cell wall have many important functions, such as providing the form of the primary cell wall as a polysaccharide matrix component, influencing secondary cell wall structure formation, and supporting cell-to-cell adhesion [30,31,32]. Pectins are especially suited for surveillance owing to the activity of pectin methylesterases (PMEs) and other pectin-modifying enzymes [33]. Due to their multitude of states and interactions, pectins are presumed to be highly responsive to the environment. In plants, pectins, as major elements of the primary cell wall, are also dynamic compounds in reaction to pathogens. The main pectin components (65%), homogalacturonans (HGs), according to Forand et al. [34], were postulated as defensive molecules in higher plants during infection and have been linked to disease resistance in many reports [2]. Moreover, the status of the methylesterification of pectins is a critical factor that directly influences cell wall structure [35]. The most important in HG methylesterification/demethylesterification are tuned by two enzymes: PME [EC 3.1.1.11] and proteinaceous PME inhibitors (PMEIs) [36,37], with a diverse effect on pectin biosynthesis. These enzymes primarily participate in stress response, signaling, and defense immunity, including in immune priming reactions [8,38,39]. Furthermore, the promotion of the PMEI that counteracts the PME improves disease resistance. According to Liu et al. [40], virus suppression of *Gossypium hirsutum* GhPMEI3 resulted in a susceptible reaction to *Verticillium* wilt disease. Other authors suggested that the native PME inhibitor AtPMEI13 can modulate the inhibitory affinity of the PME, which confirmed its defensive potential in plant–insect pathogen infestation [41]. Moreover, the screening of mutants with modification of specific cell wall polysaccharides indicated the importance of pectin modulators for penetration of resistance to *Colletotrichum* [42]. Finally, given the numerous functions of pectins and many examples of their differentially regulated modulators involved in susceptibility and/or resistance, it must be emphasized that their role is highly dependent on particular pathogen lifestyles and host plants. Therefore, our studies aimed to examine the regulation of selected *Arabidopsis thaliana* pectin metabolism components in susceptible *rbohD*–TuMV and Col-0-TuMV and resistance *rbohF*–TuMV and *rbohD/F*-TuMV reactions. This study demonstrated that cell wall rearrangement as an effect of TuMV inoculation affected PME3, PMEI2, and PMEI3, depending on the types of susceptible and resistance responses to TuMV. PME3, but not PME17, was associated with the *rbohD*–TuMV susceptible reaction leading to low/non-methylesterificated HG distribution, whereas PMEI2 and PMEI3 were important signaling factors in cell wall rebuilding and defense response in the *rbohF*–TuMV and *rbohD/F*–TuMV pathosystem.

## 2. Results

### 2.1. Virus Content and Significant Ultrastructural Apoplast Changes in TuMV Infected Col-0, rbohD, rbohF, and rbohD/F

**Figure 1:**
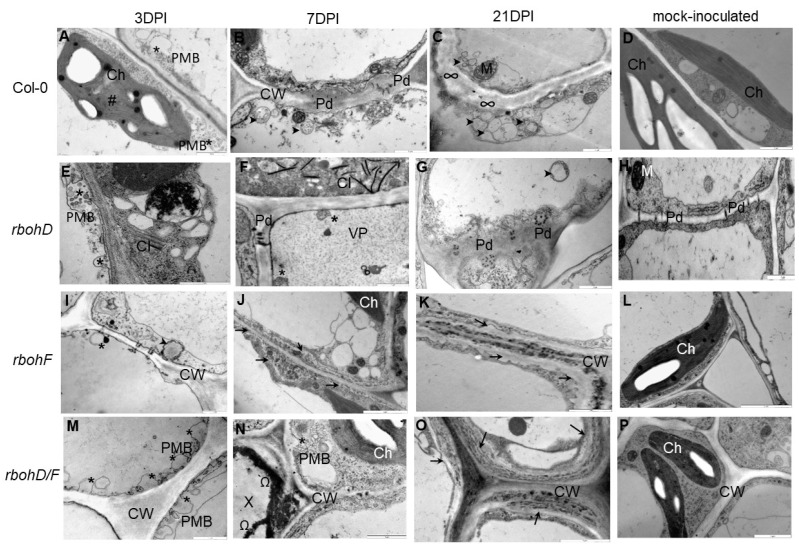
Ultrastructural changes in the apoplast area of Col-0 (**A**–**C**), *rbohD* (**E**–**G**), *rbohF* (**I**–**K**), and *rbohD/F* (**M**–**O**) plants during TuMV interaction, as well as mock-inoculated plants (**D**,**H**,**L**,**P**). (**A**) Paramural bodies (*) in palisade mesophyll cells at 3 dpi, with curved thylakoids (#) in the chloroplast (Ch). Scale bar 1 μm. (**B**) Multivesicular structures (arrowheads) and changed cell wall (CW) in the plasmodesmata (Pd) area between phloem parenchyma cells at 7 dpi. Scale bar 1 μm. (**C**) Multivesicular structures (arrowhead) and thickening of the cell wall (∞) in mesophyll cells. Scale bar 1 μm. (**D**) Noninfected mesophyll cells. Scale bar 1 μm. (**E**) Paramural bodies (*) near the cell wall in the palisade mesophyll cells, with virus cytoplasmic inclusions (CI) attached to the ER and electron-dense material inside vesicular structures. Scale bar 1 μm. (**F**) Small paramural bodies (*) in phloem sieve elements, with virus particles (VP) in sieve elements and virus cytoplasmic inclusions (CI) in phloem parenchyma cells. Scale bar 1 μm. (**G**) Multivesicular structures (arrowhead) in the vacuole of spongy mesophyll cells, along with expanded cell wall in the plasmodesmata area (Pd) 21 days after TuMV inoculation. Scale bar 1 μm. (**H**) Cell wall in noninfected phloem parenchyma cells. Scale bar 1 μm. (**I**) Paramuralar bodies (*) and multivesicular bodies (arrowhead) around the cell wall in the epidermis 3 days after TuMV inoculation. Scale bar 1 μm. (**J**) Rebuilding of the cell wall (arrow) 7 days after virus inoculation. Scale bar 2 μm. (**K**) Cell wall thickening and rebuilding (arrows) 21 days after TuMV between mesophyll cells. Scale bar 2 μm. (**L**) Noninfected mesophyll cells. Scale bar 1 μm. (**M**) Dynamic induction of paramural bodies (*) in the epidermis 3 days after TuMV. Scale bar 1 μm. (**N**) Phenolic-like compounds (Ω) inside xylem tracheary elements (X). Paramural bodies (*) in xylem parenchyma cells 7 days after virus inoculation. Scale bar 1 μm. (**O**) Rebuilt cell wall (arrows) between phloem parenchyma cells 21 dpi after TuMV inoculation. Scale bar 2 μm. (**P**) Noninfected phloem parenchyma cells. Scale bar 1 μm.

The ultrastructural analyses of the apoplast area between 3 and 21 days post-TuMV inoculation indicated significant changes compared to susceptible (*rbohD*, Col-0) (Figure 1A–C,E–G) and resistance (*rbohF*, *rbohD/F*) reactions (Figure 1I–K,M–O), as well as infected and mock-inoculated plants (Figure 1D,H,L,P). The Col-0 and mutant plants exhibited different levels of TuMV (Figure S1). Virus titers, based on the expression of the TuMV-CP gene, steadily increased in Col-0 and *rbohD* plants, whereas in *rbohF* and *rbohD/F* plants, they dynamically decreased between 3 and 21 dpi (Figure S1). In susceptible interactions, the formation of paramural bodies was observed starting from 3 dpi (Figure 1A,E). Moreover, multivesicular structures were formed from the cell wall to the vacuole between 7 and 21 dpi of virus inoculation (Figure 1B,C,F,G), accompanied by changes in cell wall structure and thickening not only around the plasmodesmata area. Dynamic formation of paramural bodies during the resistance reaction was observed between 3 and 21 dpi, especially in the epidermis (Figure 1I,M). Seven days post-TuMV inoculation, cell wall structures were rebuilt in the mesophyll as well as vascular tissues (Figure 1J,N). Furthermore, thicker cell walls than those in susceptible and mock-inoculated plants were noticed in mesophyll and vascular tissues at the 21 dpi timepoint (Figure 1K,O).

### 2.2. Crucial Modulation of Relative Expression of Selected AtPME, AtPMEI, and AtGAUT1, AtGAUT7 Genes in Resistant (rbohF and rbohD/F) and Susceptible (rbohD, Col-0) TuMV-Infected Plants

**Figure 2:**
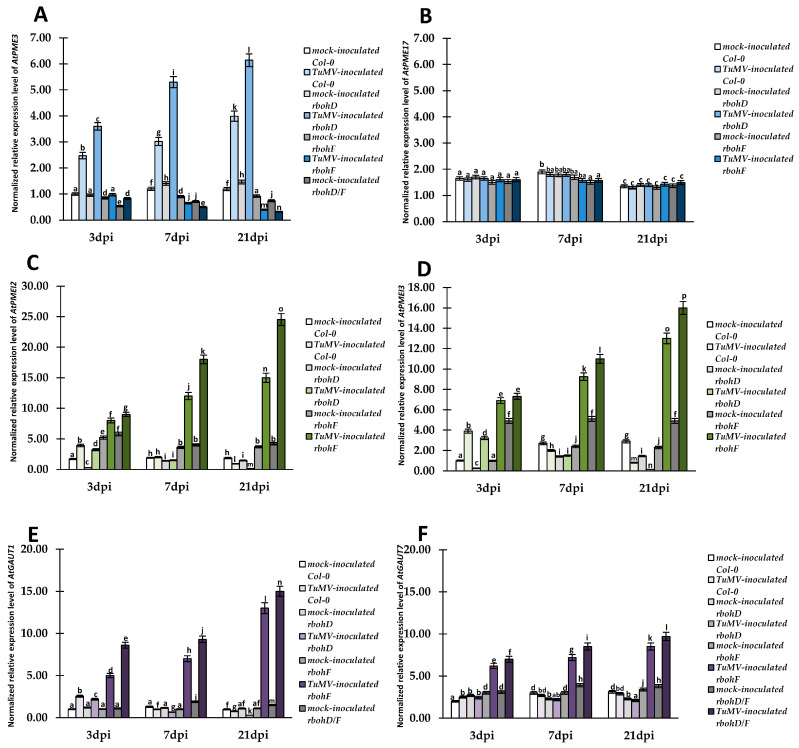
Normalized relative expression levels of *AtPME3* (**A**), *AtPME17* (**B**), *AtPMEI2* (**C**), *AtPEMI3* (**D**), *AtGAUT1* (**E**), and *AtGAUT7* (**F**), calculated based on the mean expression of *AtEf1α* and *AtF-Box* reference genes in mock-inoculated and virus-inoculated Col-0, *rbohD*, *rbohF*, and *rbohD/F* plants at 3, 7, and 21 dpi. The mean values of the normalized expression levels were calculated and analyzed using ANOVA and Tukey’s HSD test at *p* < 0.05. Statistically significant values are indicated by different letters above the bars.

Ultrastructural analyses of Col-0 and *rboh*-mutant plants infected with TuMV focused on cell wall changes, which were in line with our previous investigation on glutathione content in the apoplast [28] during different reactions to the virus. To examine the potential association of genetic factors in cell wall remodeling, we selected pectin metabolism-associated genes, such as pectin methylesterase 3 and 17 (*AtPME3* and *AtPME17*), pectin methylesterase inhibitors 2 and 3 (*AtPMEI2* and *AtPMEI3*), and galacturonosyltransferases 1 and 7 (*AtGAUT1* and *AtGAUT7*), for analysis of relative expression. In the current literature, pectin methylesterases (AtPME3, AtPME17), and methylesterase inhibitors (AtPMEI2 and AtPMEI3) were reported as key elements for susceptibility or resistance in different pathogen interactions [43,44,45,46,47]. Moreover, we also chose the *AtGAUT1* and *AtGAUT7* genes, considering that the proteins encoded by these genes are critical for HG pectin synthesis [48]. Analyses of normalized relative gene expression for all selected *Arabidopsis thaliana* genes at 3, 7, and 21 dpi were performed and indicated an interesting connection between the type of plant reaction to TuMV. *AtPME3* and *AtPME17* relative expression (Figure 2A,B) indicated statistically significant changes in TuMV-inoculated plants only for *AtPME3*. *AtPME3* was significantly upregulated in susceptible TuMV-inoculated Col-0 (1.61 fold) and *rbohD* (1.70 fold) plants between 3 to 21 dpi (Figure 2A). Moreover, *AtPME3* expression was highest in TuMV-infected *rbohD* plants. In contrast, *rbohF*-TuMV and *rbohD/F*-TuMV plants exhibited downregulation of *AtPME3* between 3 and 21 dpi, of 2.43 fold and 2.59 fold, respectively. A different trend was observed in the expression of selected *AtPMEIs* (*AtPEMI2* and *AtPMEI3*), where the expression of *AtPMEI2* and *AtPMEI3* increased between 3 to 21 dpi in virus-inoculated *rbohF* (1.88 fold for *AtPMEI2* and *AtPMEI3*) and *rbohD/F* (2.67 and 2.19 fold, respectively, for *AtPMEI2* and *AtPMEI3*) (Figure 2C,D). Moreover, the highest level of expression for both analyzed *AtPMEI*s was noticed in *rbohD/F*-TuMV. In contrast, in Col-0-TuMV and *rbohD*-TuMV, a significant decrease in *AtPMEI2* and *AtPMEI3* between 3 and 21 dpi was reported. Moreover, the highest reduction was observed for *rbohD*-TuMV plants (about 20.64 fold and 18.35 fold, respectively, for *AtPMEI2* and *AtPMEI3*). These results indicated that the upregulation of *AtPMEI2* and *AtPMEI3* was associated with an increased resistance reaction typical for *rbohF* and *rbohD/F* against TuMV. A similar connection with the resistance reaction was also shown by expression analyses of *AtGAUT1* and *AtGAUT7* (Figure 2E,F). Both of these genes were highly upregulated in *rbohF*-TuMV and *rbohD/F*-TuMV interactions between 3 and 21 dpi (Figure 2E,F). Moreover, the highest induction of 1.74 fold (for *AtGSTU1*) and 1.39 fold (in the case of *AtGSTU7*) was observed in *rbohD/F*-TuMV in contrast to susceptible Arabidopsis plants (Col-0 and *rbohD*). The *AtGAUT1* gene expression was significantly downregulated between 3 to 21 dpi in the susceptible reaction (Figure 2F). The lowest expression levels were revealed at 21 dpi in *rbohD*-TuMV, and downregulation was about 7.59 fold between 3 to 21 dpi, whereas the expression of *AtGAUT7* did not significantly change in Col-0-TuMV and *rbohD*-TuMV plants compared to mock-inoculated plants. These findings indicated that the resistance reaction against TuMV required upregulation of two AtPMEIs (*AtPMEI2* and *AtPMEI3*) and two AtGAUTs (*AtGAUT1* and *AtGAUT7*) in *rbohF* and *rbohD/F* mutants. Furthermore, the resistance reaction to TuMV was also associated with no significant change in *AtPME17* expression and downregulation of *AtPME3*. This fact may confirm that the synthesis and presence of methylesterificated homogalacturonan pectins can be important in resistant plants with control of the demethylesterification process catalyzed by *AtPME3*.

### 2.3. Subcellular Significant Localization Changes of PME3, PMEI2, PMEI3, and GAUT1 in Susceptibility and Resistance Arabidopsis Reaction to TuMV in rbohD, rbohF, and rbohD/F

Taking into account results obtained from the relative expression of selected genes associated with pectins in different reactions to TuMV, PME3, PMEI2, PMEI3, and GAUT1 proteins were localized at the ultrastructural level by quantified immunogold labeling. Considering the changes in gene expression tendency, we selected for this step 7 and 21 days past virus inoculation. Moreover, based on data presented by Atmodjo et al. [48], GAUT1 can act in plant cells with GAUT7, but our data showed that the level of *AtGAUT1* expression was more highly induced than *AtGAUT7* in resistant plants. Therefore, we decided to select GAUT1 for localization. Firstly, the potential subcellular localization was checked by using the bioinformatic server SUBA5, which connects most protein predictor localization systems and new data from mass spectrometry assay (MS/MS) to predict the potential localization of *A. thaliana* proteins. SUBA program consensus (SUBAcon) indicates potential protein localization. For PME3, PMEI2, and PMEI3, SUBAcon suggests extracellular/cell wall localization, whereas for the GAUT1 protein, the Golgi network has been indicated (Table S3). However, bioinformatic predictors connected with SUBA5 adding MS/MS data indicated that the localization of those proteins in cells can be wider than suggested only by SUBAcon (Table S3). Therefore, we performed direct immunogold localization of these proteins.

**Figure 3:**
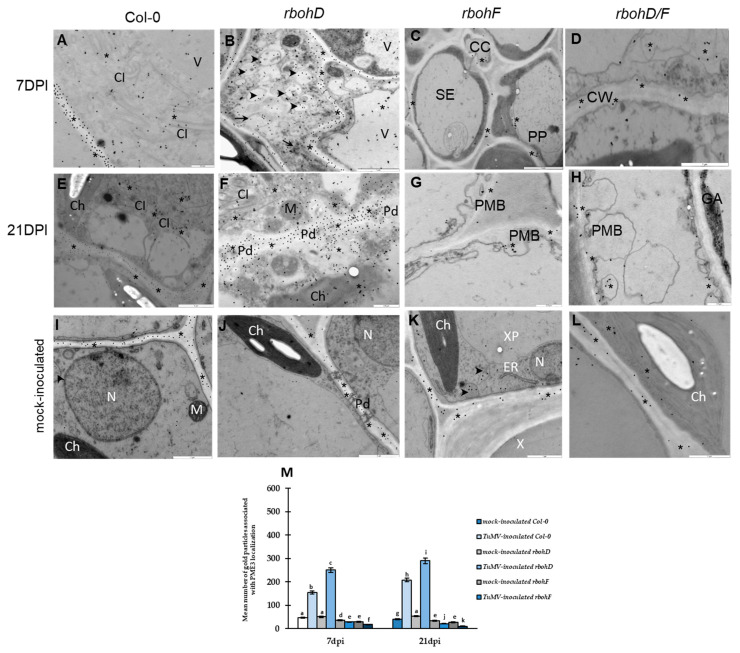
Localization (**A**–**L**) and quantification of gold particles associated with PME3 (**M**) in susceptible Col-0- (**A**,**E**), rbohD-TuMV (**B**,**F**), and resistance rbohF-TuMV (**C**–**G**) and rbohD/F-TuMV (**D**,**H**) with mock-inoculated plant (**I**–**L**) interactions. (**A**) PME3 (*) localization in the cell wall and the cytoplasm and around virus cytoplasmic inclusion (CI) in mesophyll cells. Scale bar 0.5 μm. (**B**) PME3 (*) deposition in changed cell wall, paramural bodies (PMB, arrow), and multivesicular structures (arrowhead, MVB) in phloem parenchyma cells. Weak deposition present in a vacuole (V). Scale bar 1 μm. (**C**) Weak localization in cell wall and cytoplasm of phloem cells. CC—companion cell, PP—phloem parenchyma, SE—sieve element. Scale bar 1 μm. (**D**) PME3 (*) in cell wall (CW) and cytoplasm. Scale bar 1 μm. (**E**) PME3 (*) in cell walls and around TuMV cytoplasmic inclusions (CI). Singular gold particles in chloroplast (Ch). Scale bar 1 μm. (**F**) PME3 (*) in expanded plasmodesmata (Pd) area in the cell wall, in the cytoplasm near CI, and inside chloroplast (Ch). A few particles in mitochondria (M). Scale bar 0.5 μm. (**G**) Weak PME3 (*) deposition in paramural bodies (PMB) and cell walls in the epidermis. Scale bar 0.5 μm. (**H**) PME3 in the cell wall and paramural bodies (PMB) in the epidermis. GA—Golgi network. Scale bar 0.5 μm. (**I**) PME3 (*) in the cell wall and vesicular structure (arrowhead) in mock-inoculated mesophyll cell. Ch—chloroplast, M—mitochondria, and N—nucleus. Scale bar 1 μm. (**J**) PME3 (*) in cell wall with plasmodesmata (Pd) of mock-inoculated mesophyll cells. Ch—chloroplast, N—nucleus. Scale bar 1 μm. (**K**) PME3 (*) in the cell wall of tracheary element (X) and vesicular structures (arrowhead) in mock-inoculated xylem cells. Ch—chloroplast, ER—endoplasmic reticulum, N—nucleus, XP—xylem parenchyma. Scale bar 1 μm. (**L**) PME3 (*) in the cell wall, chloroplast (Ch), and cytoplasm in mock-inoculated mesophyll cells. Scale bar 1 μm. (**M**) Quantification of gold particles associated with PME3 in *A. thaliana* in mock-inoculated and virus-inoculated Col-0, *rbohD*, *rbohF*, and *rbohD/F* plants at 7 and 21 dpi. Using ANOVA and Tukey’s HSD test, the mean number of gold particles µm^2^ of PME3 was calculated at *p* < 0.05. Statistically significant values are indicated by different letters above the bars.

The immunogold localization of PME3 revealed statistically significant differences between susceptible (Figure 3A,B,E,F,M) and resistant *rboh* plants’ reactions (Figure 3C,D,G,H,M). Moreover, in general, virus inoculation changed the PME3 deposition in Arabidopsis leaf tissues compared to the control (Figure 3I–M). However, the localization of PME3 between 7 to 21 dpi was significantly upregulated in TuMV-inoculated Col-0 (1.35 fold) and Col-0-rbohD (1.16 fold). These results are in line with relative *AtPME3* gene expression, where the most intense induction of AtPME3 was observed in *rbohD*-TuMV and Col-0-TuMV at 7 and 21 dpi (Figure 3A,B,E,F,M). Furthermore, in the susceptible reaction, PME3 was localized not only in the cell wall, as in mock-inoculated Arabidopsis leaf tissues (Figure 3I,J), but also in the cytoplasm near the virus cytoplasmic inclusion (Figure 3A,E,F). In *rbohD*, where the localization was the most intense, PME3 deposition in the rebuilt cell wall was accompanied by the location in multivesicular structures, paramural bodies, and chloroplasts with a vacuole (Figure 3B,F). In comparison, the *rbohF* and *rbohD/F* plants after virus inoculation showed a decrease in PME3 between 7 and 21 dpi, respectively, of 1.33 fold and 1.7 fold (Figure 3M). In resistant *rbohF* and *rbohD/F*, PME3 deposition was weak, and its location was noticed in paramural bodies near the rebuilt cell wall and cytoplasm (Figure 3C,D,G,H). Moreover, the weakest localization, even lower than in mock-inoculated *rbohF* and *rbohD/F* plants (Figure 3K,L), was noticed in *rbohD/F*-TuMV inoculation (Figure 3M).

**Figure 4:**
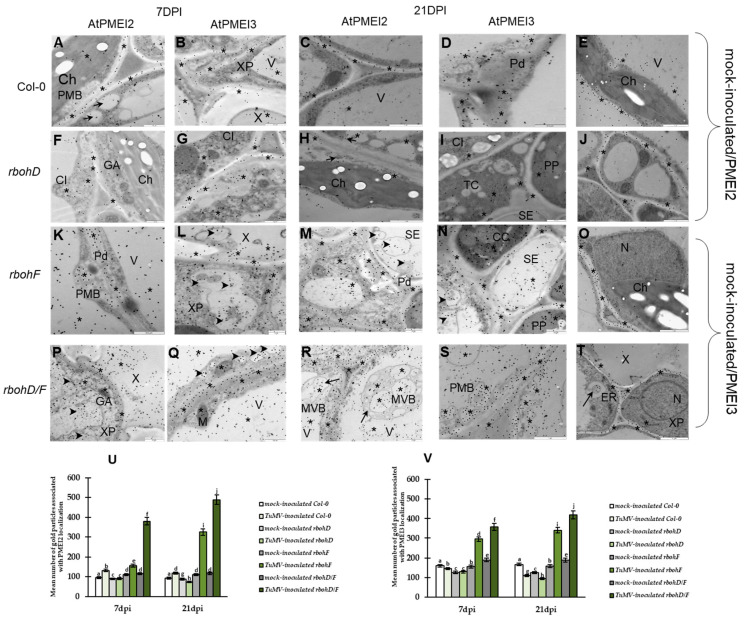
Localization (**A**–**T**) and quantification of gold particles associated with PMEI2 (**U**) and PMEI3 (**V**) in susceptible Col-0- (**A**–**D**), *rbohD*-TuMV (**F**–**I**), resistance *rbohF*- (**K**–**N**), *rbohD/F*-TuMV (**P**–**S**) interactions and mock-inoculated (**E**,**J**,**O**,**T**) plants. (**A**) PMEI2 (*) in the cell wall, cytoplasm, and vesicular structures (arrow) in mesophyll cells. Ch—chloroplast, PMB—paramular bodies. Scale bar 0.5 μm. (**B**) PMEI3 (*) in cell walls and vacuoles (V) of xylem cells. X—xylem tracheary element, XP—xylem parenchyma cells. Scale bar 0.5 μm. (**C**) PMEI2 (*) in the cell wall and vacuole of phloem parenchyma cells. Scale bar 1 μm. (**D**) PMEI3 (*) around changed cell wall with plasmodesmata (Pd). Scale bar 0.5 μm. (**E**) PMEI2 (*) in the cell wall, cytoplasm, and vacuole (V) in mock-inoculated mesophyll cells. Ch—chloroplast. Scale bar 1 μm. (**F**) PMEI2 (*) in changed cell wall, and cytoplasm near virus cytoplasmic inclusions (CI). Ch—chloroplast, GA—Golgi network. Scale bar 0.5 μm. (**G**) PMEI3 (*) in the changed cell wall and cytoplasm, also near the virus cytoplasmic inclusions (CI). Scale bar 0.5 μm. (**H**) PMEI2 (*) in the plasmalemma (arrow) and cytoplasm around the cell wall. Ch—chloroplast. Scale bar 1 μm. (**I**) PMEI3 (*) in the cytoplasm of phloem cells and around the virus cytoplasmic inclusion (CI). PP—phloem parenchyma, SE—sieve element, TC—transfer cell. Scale bar 0.5 μm. (**J**) PMEI3 (*) in the cell wall of phloem parenchyma cells. Scale bar 1 μm. (**K**) PMEI2 (*) in changed cell walls with paramular bodies (PMB) and vacuoles (V). Scale bar 0.5 μm. (**L**) PMEI3 (*) in the cell wall of xylem cells. Deposition in the vesicular structure (arrowhead) and cytoplasm in the xylem parenchyma (XP) and in the xylem tracheary element (X). Scale bar 0.5 μm. (**M**) PMEI2 (*) in the cytoplasm, with changed the cell wall between phloem cells. Deposition in membranous structures (arrowhead) in the sieve element (SE). Scale bar 0.5 μm. (**N**) PMEI3 (*) in the cell wall, inside the sieve element (SE), and necrotized phloem parenchyma (PP) and companion cell (CC). Localization in the vesicular structure (arrowhead) in phloem parenchyma. Scale bar 0.5 μm. (**O**) PMEI2 (*) in the cell wall of a mock-inoculated mesophyll cell. Ch—chloroplast, N—nucleus. Scale bar 1 μm. (**P**) PMEI2 (*) inside xylem tracheary elements (X). Deposition in the cytoplasm, Golgi network (GA), and vesicular structures (arrowhead) of a xylem parenchyma cell (XP). Scale bar 0.5 μm. (**Q**) PMEI3 (*) in the cell wall with vesicles around (arrowhead) and in the cytoplasm of the mesophyll cell. Localization is also presented in the vacuole (V) and mitochondria (M). Scale bar 0.5 μm. (**R**) PMEI2 (*) in the multivesicular bodies (arrows, MVB) and the cell wall of phloem parenchyma (PP) cells. Deposition is also in the cytoplasm and vacuole. Scale bar 0.5 μm. (**S**) PMEI3 (*) deposition in the cell wall and paramural bodies (PMB) of the epidermis cell. Scale bar 1 μm. (**T**) PMEI3 (*) deposition in the cell walls of xylem cells. X—xylem tracheary element, XP—xylem parenchyma. A few gold particles are also in the endoplasmic reticulum (ER) and vesicular structures (arrow). N—nucleus. Scale bar 1 μm. (**U**) Quantification of gold particles associated with PMEI2 in *A. thaliana* in mock-inoculated and virus-inoculated Col-0, *rbohD*, *rbohF*, and *rbohD/F* plants at 7 and 21 dpi. (**V**) Quantification of gold particles associated with PMEI3 in *A. thaliana* in mock-inoculated and virus-inoculated Col-0, *rbohD*, *rbohF*, and *rbohD/F* plants at 7 and 21 dpi. Using ANOVA and Tukey’s HSD test, the mean number of gold particles µm^2^ of PMEI2 (**U**) and PMEI3 (**V**) was calculated at *p* < 0.05. Statistically significant values are indicated by different letters above the bars.

On the other hand, downregulation of PMEI2 and PMEI3 deposition between 7 and 21 dpi (Figure 4A–D,F–I,U,V) was revealed in Col-0 and *rbohD* plants. PMEI2 and PMEI3 were localized in the cell wall with induced paramural bodies, as well as in the cytoplasm and vacuole (Figure 4A,B,F,G). However, at the 21 dpi timepoint, they were mainly located around the cell wall area in the mesophyll or vascular tissue (Figure 4C,D,H,I). Moreover, the highest reduction was characterized for *rbohD* plants with the virus at 21 dpi (about 1.23 fold and 1.37 fold, respectively, for PMEI2 and PMEI3) (Figure 4F–I,U,V), and the localization at this timepoint was sometimes even lower compared to mock-inoculated *rbohD* or Col-0 (Figure 4C–E,H–J). In contrast, PMEI2 and PMEI3 were both upregulated in virus-inoculated *rbohF* (2.08 fold and 1.15 fold, respectively, for PMEI2 and PMEI3) and *rbohD/F* (1.29 and 1.17 fold, respectively) between 7 and 21 dpi (Figure 4U,V). Moreover, both inhibitors’ epitopes were deposited in the cell walls with paramural bodies (also with plasmodesmata) and decorated multivesicular and membranous structures in the cytoplasm, as well as ER, Golgi network, and vacuoles in all leaf tissues (Figure 4K–N,P–S). Furthermore, the localization was more intense at 21 dpi than at 7 dpi after TuMV inoculation, as well as compared with mock-inoculated tissues (Figure 4K–T). The most intense localization level of epitopes of PMEI2 and PMEI3 was observed in *rbohD/F* plants with TuMV (Figure 4P–S,U,V).

These results strictly correspond with the relative gene expression, suggesting that the upregulation of the genes/proteins *AtPMEI2*/PMEI2 and *AtPMEI3*/PMEI3 was associated with increased resistance and the resistance reaction of *rbohF* and *rbohD/F* against TuMV.

**Figure 5:**
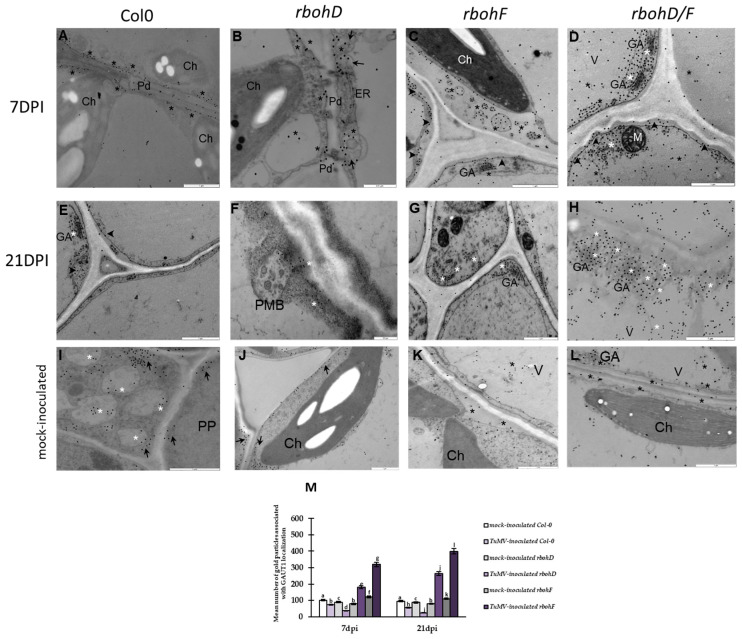
Localization (**A**–**L**) and quantification (**M**) of gold particles associated with GAUT1 in susceptible Col-0- (**A**,**E**), *rbohD*-TuMV (**B**,**F**), resistance *rbohF*-TuMV (**C**,**G**), rbohD/F-TuMV (**D**,**H**) interactions and mock-inoculated (**I**–**L**) plants. (**A**) GAUT1 (*) in the plasmalemma, with a few gold particles in the cell wall near plasmodesmata (Pd) in the mesophyll cell. Ch—chloroplast. Scale bar 1 μm. (**B**) GAUT1 (*) in the plasmalemma and vesicular structure (arrows) near plasmodesmata (Pd) in a mesophyll cell. Ch—chloroplast, ER—endoplasmic reticulum. Scale bar 0.5 μm. (**C**) GAUT1 (*) in the vesicles, Golgi network (GA), and plasmalemma in the mesophyll. Ch—chloroplast. Scale bar 1 μm. (**D**) GAUT1 (*) in the Golgi network (GA), between the plasmalemma and cell wall (arrowheads). Deposition also in the vacuole (V). M—mitochondria. Scale bar 1 μm. (**E**) GAUT1 (*) in the Golgi network (GA) and between the plasmalemma and cell wall (arrowhead). Scale bar 1 μm. (**F**) GAUT1 (*) in paramural bodies (PMB), near changed cell wall between mesophyll cells. Scale bar 0.5 μm. (**G**) GAUT1 (*) in the Golgi network (GA) and cytoplasm of a phloem parenchyma (PP) cell. A few gold particles in the cell wall. M—mitochondria, V—vacuole. Scale bar 1 μm. (**H**) GAUT1 localization in the Golgi network (GA) and plasmalemma near changed cell wall. Deposition in the vacuole (V) of a mesophyll cell. Scale bar 1 μm. (**I**) GAUT1 (*) in vesicular structures/small vacuoles (V) and near the cell wall (arrows) of phloem parenchyma (PP) cells. Scale bar 1 μm. (**J**) GAUT1 (*) along the plasmalemma (arrows) in mesophyll cells. Ch—chloroplast. Scale bar 1 μm. (**K**) GAUT1 (*) between the plasmalemma and cell wall and in the cytoplasm of mesophyll cells. A few gold particles are in the vacuole (V). Ch—chloroplast. Scale bar 1 μm. (**L**) GAUT1 (*) in the Golgi network (GA), near the plasmalemma along the cell wall in mesophyll cells. A few gold particles are in the vacuole (V). Ch—chloroplast. Scale bar 1 μm. (**M**) Quantification of gold particles associated with GAUT1 in *A. thaliana* in mock- and virus-inoculated Col-0, *rbohD*, *rbohF*, and *rbohD/F* plants at 7 and 21 dpi. Using ANOVA and Tukey’s HSD test, the mean number of gold particles µm^2^ of GAUT1 was calculated at *p* < 0.05. Statistically significant values are indicated by different letters above the bars.

To evaluate *A. thaliana* GAUT1, which is responsible for HGs synthesis, immunogold labeling localization during TuMV infection at 7 and 21 dpi timepoints, when the *AtGAUT1* relative gene expression was the most induced, was conducted. In mock-inoculated Col-0 and *rboh*-mutant plants, GAUT1 was observed in the plasma membrane, Golgi network, and vacuole (Figure 5). The GAUT1 protein deposition was induced in virus-inoculated *rbohF* and *rbohD/F* plants between 7 and 21 dpi compared to mock-inoculated plants, as well as to susceptible Col-0 and *rbohD* (Figure 5A–M). The highest induction (1.25 fold) was observed in TuMV-*rbohD/F* (Figure 5D,H,M). Moreover, the GAUT1 in resistant TuMV-*rbohF* and TuMV-*rbohD/F* was mainly localized in the trans-Golgi network and plasmalemma with vesicular/membranous structures (Figure 5C,D,G,H). On the other hand, the plants with susceptibility, Col-0 and *rbohD*, reacted with downregulation of GAUT1 epitope localization (Figure 5A,B,E,F,M). Moreover, the lowest localization of GAUT1 was reported at 21 dpi in TuMV-*rbohD* plants, and the reduction was about 1.54 fold between 7 and 21 dpi. The GAUT1 location documented in TuMV-Col-0 and TuMV-*rbohD* between 7 and 21 dpi was even less intense than in mock-inoculated Col-0 and *rbohD* plants. Furthermore, GAUT1 in the susceptible reaction was deposited along with the plasmalemma, in the cytoplasm near the cell wall and plasmodesmata (Figure 5A,B,E,F). The obtained results suggested that the induction of relative *AtGAUT1* and *AtGAUT7* gene expression corresponded with Arabidopsis GAUT1 protein deposition and was related to both the resistance interactions: *rbohF*-TuMV and especially *rbohD/F*-TuMV. 

### 2.4. Highly Methylesterificated HGs’ Domination in Resistance Reactions to TuMV

**Figure 6:**
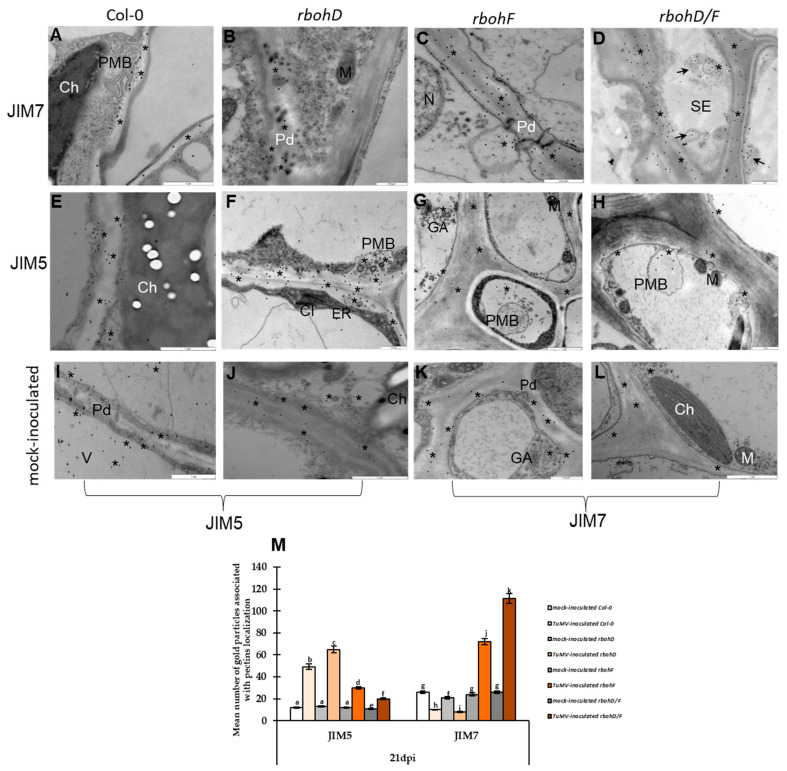
Localization (**A**–**L**) and quantification (**M**) of low/non-methylesterificated homogalacturonan (detected by JIM5) and highly methylesterificated homogalacturonan (detected by JIM7) in susceptible Col-0- (**A**,**E**), *rbohD*-TuMV (**B**,**F**) interaction, and resistant *rbohF*- (**C**,**G**), *rbohD/F*-TuMV (**D**,**H**) interaction with mock-inoculated (**I**–**L**) plants at 21 days after virus inoculation. (**A**) Highly methylesterificated HGs (*) along the cell wall in the paramural bodies (PMB) forming area in a palisade mesophyll cell. Ch—chloroplast. Scale bar 1 μm. (**B**) Highly methylesterificated HGs (*) in the cell wall plasmodesmata (Pd) area. M—mitochondrion. Scale bar 0.5 μm. (**C**) Highly methylesterificated HGs (*) in the cell wall and vesicular structure next to plasmodesmata (Pd) in mesophyll cells. N—nucleus. Scale bar 0.5 μm. (**D**) Highly methylesterificated HGs (*) in cell walls and multivesicular structures (arrows) in phloem cells. SE—sieve element. Scale bar 1 μm. (**E**) Low/non-methylesterificated HGs (*) along the plasmalemma and cell wall in a mesophyll cell. Ch-chloroplast. Scale bar 1 μm. (**F**) Low/non-methylesterificated HGs (*) in changed cell walls and paramural bodies (PMB) between mesophyll cells. CI—virus cytoplasmic inclusion, ER—endoplasmic reticulum. Scale bar 0.5 μm. (**G**) Low/non-methylesterificated HGs (*) in the Golgi network and cell wall in phloem cells. M—mitochondria, PMB—paramural bodies. Scale bar 1 μm. (**H**) Low/non-methylesterificated HGs (*) in paramural bodies (PMB) with a few gold particles in the cell walls in phloem cells. M—mitochondria. Scale bar 1 μm. (**I**) Low/non-methylesterificated HGs (*) around the cell wall with plasmodesmata (Pd) and in a vacuole (V) in a mock-inoculated mesophyll cell. Scale bar 1 μm. (**J**) Low/non-methylesterificated HGs (*) in the cell walls and vesicles in mock-inoculated mesophyll cells. Ch—chloroplast. Scale bar 1 μm. (**K**) Highly methylesterificated HGs (*) in the cell wall between mock-inoculated phloem cells. GA—Golgi network, M—mitochondria, Pd—plasmodesmata. Scale bar 0.5 μm. (**L**) Highly methylesterificated HGs (*) in the cell wall between mock-inoculated mesophyll cells. Ch—chloroplast, M—mitochondrion. Scale bar 0.5 μm. (**M**) Quantification of gold particles associated with low/non-methylesterificated HGs (JIM5) and highly methylesterificated HGs (JIM7) detected in *Arabidopsis thaliana* in mock-inoculated and virus-inoculated Col-0, *rbohD, rbohF*, and *rbohD/F* plants at 21 dpi. Using ANOVA and Tukey’s HSD test, the mean number of gold particles µm^2^ was calculated at *p* < 0.05. Statistically significant values are indicated by different letters above the bars.

In the analysis of PME activity and its correlation with cell wall methylester levels during susceptible Col-0-TuMV, *rbohD*-TuMV, and resistance *rbohF*-TuMV, *rbohD/F*-TuMV indicated higher demethylesterification activity in susceptible Col-0-TuMV, and especially *rbohD*-TuMV interactions. The highest level of PME activity was detected at 21 days post-inoculation with TuMV in both susceptible interactions. We checked the tendency in localization between high and low/non-methylesterificated HGs expressed through JIM7 and JIM5 detection during both types of reaction to TuMV. The immunogold localization (Figure 6A–L) confirmed by quantification (Figure 6M) indicated that low/non-methylesterificated HGs were deposited more intensely during susceptible TuMV interactions with *rbohD* mutant and Col-0 plants compared to both resistance types of interaction (*rbohF*, *rbohD/F*) as well as mock-inoculated plants (Figure 6E–J). These low/non-methylesterificated HG pectins were found in the changing cell wall, near the plasmalemma and plasmodesmata in *rbohD* and Col-0 plants infected with TuMV. In contrast, in resistance interactions with *rbohF* and *rbohD/F* mutants, the most intense deposition was observed for highly methylesterificated HGs compared to mock-inoculated plants (Figure 6A–D,K,L). These highly methylesterificated HGs were located near the plasmalemma with paramural bodies and in multivesicular structures (Figure 6C,D,G,H). These findings suggest that demethylesterification of HG pectins is closely associated with susceptible interactions, especially in *rbohD* mutants and Col-0 plants, while intense accumulation of highly methylesterificated HG pectins is characteristic of resistance interactions with *rbohD/F* and *rbohF* mutant plants.

### 2.5. Induction of PME Activity in Susceptible Interaction with a Decrease of Cell Wall Methylesters Level

**Figure 7:**
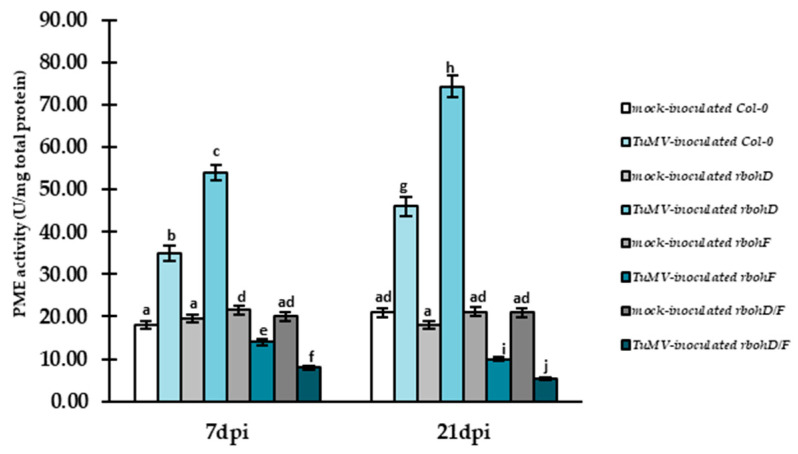
Estimation of PME activity in mock-inoculated and virus-inoculated Col-0, *rbohD*, *rbohF*, and *rbohD/F* leaves at 7 and 21 dpi. The mean activities (in U/mg total protein) were calculated. Significant differences between classes at the *p* < 0.05 level were assessed by ANOVA with post hoc Tukey’s HSD. Statistically significant values are indicated by letters above the chart bars.

**Figure 8:**
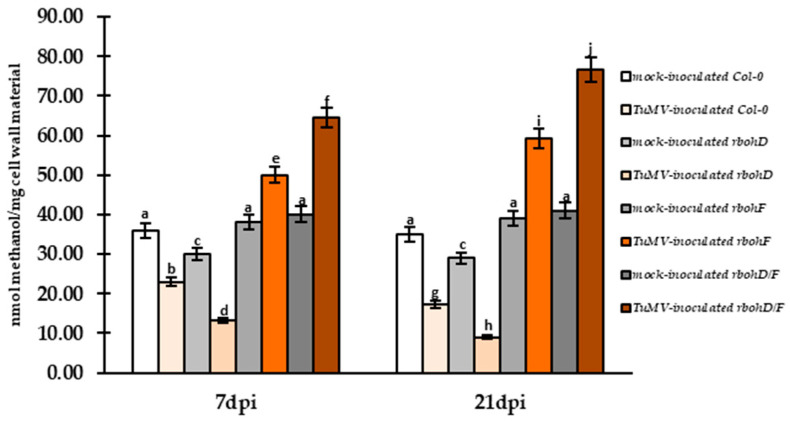
Quantification of methylesters in mock-inoculated and virus-inoculated Col-0, *rbohD, rbohF, and rbohD/F* leaves at 7 and 21 dpi. Estimated methylester content as based on methanol associated with saponified cell wall materials in leaves. Significant differences between classes at the *p* < 0.05 level were assessed by ANOVA with post hoc Tukey’s HSD. Statistically significant values are indicated by letters above the chart bars.

Validation of PME activity revealed a significant increase between 7 and 21 dpi in virus-inoculated susceptible (Col-0) and increased susceptibility plants (*rbohD*), partially corresponding to increased expression of *AtPME3*. The activity increased by 1.31 fold and 1.37 fold for Col-0 and *rbohD* plants with the virus between 7 and 21 dpi (Figure 7). In contrast, PME activity was downregulated in virus-inoculated *rbohF* and *rbohD/F* plants between 7 and 21 dpi, with the lowest activity observed in *rbohD/F* plants with the virus at 21 dpi (Figure 7). This trend corresponded with the estimation of cell wall methylester levels (Figure 8), which increased in plants showing an increased resistance reaction (*rbohF*, *rbohD/F*) to TuMV between 7 and 21 dpi, with the highest level in *rbohD/F*-TuMV plants (1.19 fold) (Figure 8). In contrast, Col-0 and *rbohD*-TuMV plants exhibited downregulation of methylester levels, with the highest reduction occurring in *rbohD*-TuMV (1.47 fold) between 7 and 21 dpi (Figure 8). These results suggest that the demethylesterification process is associated with the susceptible reaction of *rbohD* and Col-0 plants to TuMV.

## 3. Discussion

NADPH oxidases in plants can regulate or modulate responses to various plant–pathogen interactions [49]. Previous findings have shown varied reactions of *rbohD*, *rbohF*, and *rbohD/F* transposon mutants to TuMV infection [27]. Systemic TuMV infection was promoted in *rbohD* mutants, indicating that RbohD is important in TuMV infection. Moreover, virus concentration was significantly induced in *rbohD* mutants more dynamically than in Col-0 plants (Figure S1), and it was also shown by [27]. Furthermore, *rbohD*-TuMV plants displayed reduced ROS, decreased glutathione content, and higher levels of PR-1 proteins, which can act as markers of TuMV infection in this pathosystem [27,28]. Conversely, *rbohF* and *rbohD/F* mutants exhibited a significant reduction in TuMV infection accompanied by H_2_O_2_ induction compared to mock-inoculated plants. These results are compatible with a significantly dynamic increase in total glutathione content in cells and the apoplast, along with upregulation of GGT enzyme activity [28]. This observation suggests that RbohF is important in TuMV infection or may even increase susceptibility to this virus. Ultrastructural analyses of *rbohD*, *rbohF*, and *rbohD/F* interactions with TuMV have focused on changes in the plant cell wall. Previous research has mainly concentrated on cell wall changes in plant–pathogen interactions involving bacteria and fungi as mechanical and enzymatic destructors of the host apoplast [50,51,52]. However, proteins and enzymes involved in active cell wall defense responses during plant virus infection may play an important function [14,18]. Research on potato plants with different levels of resistance to PVY^NTN^ has shown that virus infection induces active cell wall rearrangements during compatible and incompatible interactions [24]. It was revealed that β-1,3-glucanase, cellulose synthase catalytic subunit CesaA4, and hydroxyproline-rich glycoproteins A4 (StEXTA4-HRGP) with potato expansin A3 (StEXPA3) are actively modulated as an effect of PVY^NTN^ inoculation [24,26]. Given these findings and the observed cell wall rearrangements during the reaction of *rbohD* and *rbohF* mutants to TuMV, we decided to explore in more detail selected components of pectin metabolism in the *rboh*-TuMV pathosystem.

Ultrastructural analysis of susceptible *rbohD* and Col-0 interactions with TuMV documented the active formation of paramural bodies in mesophyll and vascular tissues during systemic infection. Additionally, a more expanded cell wall structure was observed, especially in the plasmodesmata area, accompanied by the formation of multivesicular structures and the presence of virus particles and virus cytoplasmic inclusions. These observations are consistent with other pathosystems where cell wall alterations lead to the formation of paramural bodies attached to the plasma membrane [53,54]. Cytological modifications also include the formation of vesicles and tubules originating from the border cell wall and incorporating into the plasmalemma, as postulated in potato virus Y NTN (PVY^NTN^) and potato virus M (PVM) infections [24,55,56]. In contrast, the resistance reactions of *rbohF* and *rbohD/F* mutants to TuMV displayed significant cell wall rebuilding. Paramural bodies were forming, especially in the epidermis. The cell wall in the resistance reaction was visibly thicker in mesophyll and phloem cells, accompanied by the deposition of phenolic-like compounds in xylem tracheary elements. These ultrastructural observations are in accordance with alterations induced by *Botrytis cinerea*, *Colletotrichum*, or *Blumeria*, where phenolic substances accumulate around cell walls and may act as a barrier for pathogens [53,57,58]. Moreover, similar cell wall thickening in resistance reactions was documented during PVY^NTN^ and PVM–potato interactions [24,26,55]. Furthermore, according to data presented by O’Brien et al. [59] and Tse et al. [60], the strengthening of the plant cell wall plays a key role in plant defense and could be accompanied by the induction of wall material-associated vesicles, such as paramural bodies or multivesicular structures. Conversely, as postulated by Tse et al. [60], these structures can be arranged in degradation in the vacuole or are fused to the plasmalemma to release vesicles. Plant cell wall remodeling is a dynamic process in different interactions as well as in normal growth and development [61]. It is a well-known fact that the plant cell wall is the first barrier against pathogens, and its integrity plays an important role in plant defense [62]. Moreover, the remodeling of cell wall components is a response to exposure to biotic stress [63]. Transcriptomic profiling and microarray analyses of gene expression in susceptible and resistance responses to some groups of viruses have reported that cell wall-related genes and transcripts are regulated during plant–virus infection [15,16,17,64].

Pectins are major components of the primary cell walls of dicotyledonous and non-gramineous monocotyledonous plants, accounting for about 35% of total cell wall polysaccharides [65]. Pectins also act as active molecules in many cellular metabolism pathways, physiological processes, and signaling processes [66,67]. There are mainly four different types of pectin polysaccharides; the major ones are HGs, rhamnogalacturonans I (RGsI), the most structurally diverse rhamnogalacturonans-II (RGsII), and covalently linked RG-I xylogalacturonans (XGas) [68]. However, HGs are the most abundant pectins, constituting 65%, and recent findings underline the importance of pectins as defensive components in plants during pathogen infection [21,37,69]. HGs are synthesized in the Golgi network from nucleotide sugars and are secreted in a methylesterificated form into the cell wall [70]. Their structure can be modified by homogalacturonan-modifying enzymes (HGMEs) [71], such as pectin methylesterases (PME) [E.C. 3.1.1.11]. In Arabidopsis, PMEs belong to multigenic (66 *A. thaliana* genes) and super-multiple enzyme families [72]. PMEs selectively catalyze the process of demethylesterification in HGs and can lead to stiffness or softness of the cell wall-pectin matrix [38,73]. However, as postulated by Coculo et al. [74], the generation of low degrees of methylesterificated HG pectins may promote proton-releasing and depolymerization conducted by enzymes such as polygalacturonases (PGs) and pectate lyase-like proteins (PLLs), resulting in cell wall loosening and expansion. Namely, it was documented first by Juge et al. [75] that PME activity is modulated by a family of proteinaceous inhibitors known as PMEIs, which belong to multigenic families in Arabidopsis (77 putative PMEI genes). Therefore, it can be assumed in line with Hocq et al. [73] that PMEs with PMEI enzymes exhibit diverse effects on physiology and pectin biosynthesis. Moreover, the specific PME–PMEI relation is a critical factor in the fine-tuned degree of demethylesterificated HG pectins and that aspect may determine cell wall remodeling and integrity not only during the biological process, but also in plant–pathogen interactions [39]. Based on those statements and taking into account that other genes/enzymes related to pectin metabolism (such as PG), PLL, as well as pectin acetylesterase PAE [E.C. 3.1.1.6] were shown to be rather not induced by plant viruses or endosymbionts [70]. Therefore, we decided to examine normalized relative gene expression of selected PME and PMEI during *rboh*-TuMV interactions. Our findings indicated that *AtPME3* was highly induced in the susceptible Col-0-TuMV interaction. However, in the *rbohD*-TuMV interaction, the upregulation of *AtPME3* was the most intense. Conversely, regardless of the interaction type, *AtPME17* gene expression was unchanged compared to mock-inoculated plants. *AtPME17* has been implicated in interactions in different pathosystems [44,74]. Interestingly, Arabidopsis knockout mutant pme17 modified the trophic behavior of *Myzus persica* [70]. However, *PME17* expression is significantly increased with other aphids and whiteflies [76,77]. Furthermore, it was postulated that PME17 could participate in facilitating the progression of the stylet in wild-type plants. It seems that *AtPME17* is not engaged in the response to TuMV, whereas *AtPME3* may be responsible for pectin demethylesterification during susceptible interactions with TuMV in *rbohD* and Col-0 plants.

Consequently, the highest induced PME activity was revealed in *rbohD*-TuMV and Col-0-TuMV compared to mock-inoculated and resistant *rbohF*-TuMV and *rbohD/F*-TuMV plants. In Arabidopsis, some PME isoforms show altered expression in response to *Botrytis cinerea* [78]. Specifically, *AtPME3* is induced during infection with *B. cinerea* and *P. carotonorum*, acting as a susceptibility factor [43]. Furthermore, the expression of PME genes and PME activity in plant–virus interactions has broader implications. For example, the *StPME* gene in potato Igor was upregulated as a result of PVY^NTN^ inoculation, but it was estimated only at 0.5 h post-inoculation [79]. It is in line with our observation, where the susceptible reaction to TuMV induced *AtPME3* gene expression. Conversely, *AtPME3* was downregulated during interaction with CaLCuV at 12 dpi [80]. Additionally, Yang et al. [81] indicated that the *AtPME3* gene in TuMV-Arabidopsis was downregulated in tissues from the inoculation point (zone 0), while induction of *AtPME3* was observed millimeters away from the inoculation point, in zone 3. In tobamovirus interactions, PME proteins actively participate in TMV local transport through a requirement for virus plasmodesmata-exploring movement [82]. Specifically, the binding of TMV MP may interfere with PME activity, modifying the cell wall ion balance, inducing changes in plasmodesmata permeability, and facilitating cell-to-cell movement [83]. Moreover, Chen and Citovsky [84] have indicated that PME is also involved in TMV systemic movement, aiding in the virus’s exit from the phloem to adjacent tissues of noninfected parts of plants.

Recent reports have highlighted the stimulation of expression of multiple methanol-inducible genes (MIGs), which affects the permeability of plasmodesmata and is a consequence of pectin demethylesterification [85,86,87]. It has also been highlighted by Andika et al. [88] that PME interacts with turnip vein clearing virus (TVCV), cauliflower mosaic virus (CaMV), and even Chinese wheat mosaic virus (CWMV) at the cell wall, and the results of these interactions can be essential for virus cell-to-cell movement. PME silencing or overexpression of PMEI in Nicotiana has been shown to delay TMV and TVCV systemic movements and significantly reduce host susceptibility [89,90]. Similar to our observations in the *rbohD*-TuMV and Col-0-TuMV interactions, Dorokhov et al. [91] reported that a cell wall enriched in pectin can be more flexible and may allow dynamic changes in plasmodesmata structure [92,93,94]. Moreover, PME is involved in HG demethylesterification *in muro* accumulated in the cell wall near plasmodesmata [87,95]. Consequently, pectin demethylesterification expressed through *AtPME3* upregulation and PME activity induction, with PME3 deposition on an ultrastructural level like in *rbohD*-TuMV, seems to influence cell wall expansion changes in the plasmodesmata area, leading to virus transport from cell to cell, including to vascular tissues. In susceptible interactions with *rbohD* and Col-0-TuMV, a natural consequence of induced *AtPME3* gene expression and PME activity was the intense and statistically significant deposition of PME3 in rebuilt cell walls and multivesicular bodies compared to mock-inoculated Arabidopsis leaf tissue, and compared to resistance reactions *rbohF*-TuMV and *rbohD/F*-TuMV, has been observed. Interestingly, potyvirus cytoplasmic inclusion (CI) proteins can act as structures docking movement complexes to plasmodesmata [96,97]. Moreover, CI and the viral protein P3N-PIPO complex coordinate the formation of PD-associated structures that facilitate intercellular movement. The localization of PME3 documented during TuMV stress in the plasma membrane and vesicular structures is partly similar to observations presented by Morvan et al. [95] based on Linum PME in cortical tissue. Derksen et al. [98] and Morvan et al. [95] reported PME epitopes distributed along the plasmalemma and used antibodies that recognized epitopes of PME precursors in the Golgi network. The authors underlined that PME can be retained at the plasma membrane and also secreted into vesicular structures.

The detection of low/non-methylesterificated and highly methylesterificated HG fractions of pectins indicated that in the susceptible reactions of *rbohD* and Col-0 plants to TuMV, there was statistically significant domination of low/non-methylesterificated HGs. This was confirmed by the quantification of immunogold labeling. These low/non-methylesterificated HGs associated with the susceptible reaction to TuMV were located in the changed cell wall, as well as in the plasmalemma and near plasmodesmata. Moreover, the observation indicated that PME3 labeling in the *rbohD*- and Col-0-TuMV interactions are correlated with those obtained with low/non-methylesterificated HGs (JIM5), although the quantification indicated much more intense deposition of PME3. Generally, different authors have postulated that the deposition of HGs (detected by JIM5 and JIM7) is consistent with a weak signal [99,100]. According to Libermann et al. [101], low methylesterificated pectins through JIM5 detection in mung bean hypocotyl were documented in the Golgi network and vesicular structures. Our findings regarding the susceptible *rbohD*-TuMV and Col-0-TuMV interaction are in line with statements presented by Lionetti et al. [47] that the demethylesterification process of HGs by PME activity might result in a decrease in cell wall strength, acting as a barrier that favors virus pathogen invasion. Quite interesting observations were documented by Fan et al. [102], who found that a high abundance of demethylesterificated HGs and highly demethylesterificated pectins were correlated with low pathogenicity in bananas during infection with *Fusarium oxysporum* f. sp. cubense pathogenic races 1 (Foc1) and 4 (Foc4) on banana (Musa AAA, Brazilian). Furthermore, during interactions between host and pathogen, some authors have reported a decrease in HGs recognized by JIM5 and JIM7, and this process is involved in the response of most plants to pathogens [100,102,103]. However, opposite relationships have also been presented [104,105]. Therefore, it can be assumed that plants finely tune pectin methylesterification levels and regulate PME activity during their development. In plant–pathogen interactions, the cooperation between PME and PMEI with the degree of HGs methylesterification is associated with plant resistance to pathogens [45,106].

In general, the degree of methylesterification of HGs can play a role in determining the biomechanical properties of the cell wall [107,108]. Demethylesterification can result in forming calcium bands with other HG molecules, leading to an egg-box structure that underlies the core of the pectin gel formation process [109]. Calcium-linked HG increases cell wall hydration [110]. Moreover, Tibbits et al. [111] postulated that the strength of pectin gels is highly related to the free calcium ions in the apoplast, and the stiffness of this kind of gel can be reduced through the disassociation of calcium crosslinks. Conversely, partially demethylesterificated HGs can be easily targeted by pectin-degrading enzymes (like PG or PLL), which is especially important in bacteria and fungi interaction [72]. Otherwise, PMEI coordinated with PME is a crucial factor in regulating effects on cell wall structure properties, such as strengthening, loosening, and modulation response to virus infection, especially immune response. Our findings indicated that resistance reactions with reduction of virus content and cell wall rearranged structure in *rbohF* and *rbohD/F* to TuMV were associated with significant induction of two examined *Arabidopsis PMEI* genes: *AtPMEI2* and *AtPMEI3* (between 3 and 21 dpi). Moreover, the normalized relative gene expression of *AtPMEI2* and *AtPMEI3* was confirmed by dynamically increased PMEI2 and PMEI3 protein deposition at the ultrastructural level. Furthermore, the highest induction of both gene expression and protein deposition was reported in the *rbohD/F*-TuMV reaction. That tendency in *rbohD/F*–TuMV and *rbohF*–TuMV interaction was also accompanied by an intense decrease in PME activity. These results seem to be partly in line with a completely different pathosystem, *Arabidopsis thaliana* with *Botrytis cinerea*, presented by Lionetti et al. [45], where expression of *AtPMEI1* and *AtPMEI2* downregulated PME activity. However, in contrast to our observation, reduced PME activity resulted in an increased level of demethylesterification of HGs. It should be highlighted that pathogen secretion of cell-wall-degrading enzymes, which hydrolyze pectins, is an important step to successful plant infection by bacteria or fungi, but not for viruses [43,112,113]. Factually, plant-induced PME activity and lower levels of pectin methylesterification were observed in these interactions correlated with reduced demethylesterification of pectins like necrotrophic pathogens and/or *Pseudomonas syringae* [43,105]. Moreover, Lionetti et al. [47] postulated that upregulation of *AtPMEI10*, *AtPMEI11*, and *AtPMEI12* were characterized for response to *B. cinerea* infection, whereas *pmei10*, *pmei11*, and *pmei12* mutants displayed an increase in PME activity and decreased demethylesterification of pectins, which led to increased lesion formation during Botrytis infection. The tendency observed by Lionetti et al. [47] and Coculo and Lionetti [38] indicated that plants modulated PME activity by the expression of PMEI in response to infection. Moreover, it was pointed out that AtPMEI10, AtPMEI11, and AtPMEI12 can act as mediators of cell wall-induced plant immunity [38]. Furthermore, it was documented that PMEI can play a role as an antimicrobial factor/protein against *Fusarium*, *Alternaria brassicicola*, *Xanthomonas campestris*, or *Pseudomonas syringae* [114]. On the other hand, overexpression of *PMEI1* and *PMEI2* in Arabidopsis displayed resistance to powdery mildew and soft rot disease [45]. It has been shown that overexpression and high induction of PMEI enhanced plant resistance in different pathosystems [40,105]. According to presented statements, overexpression of PMEIs in Nicotiana as well as in Arabidopsis counteracts PMEs, leading to increased resistance to tobacco mosaic virus and turnip vein-clearing virus infection [46]. From these statements and obtained results in *rbohF*-TuMV and *rbohD/F*-TuMV pathosystems, it can be concluded that an increase of PMEI can influence reducing virus transport through downregulation of PME activity and even by hindering the enlargement of plasmodesmata.

In addition to PME–PMEI regulation, *rbohF*-TuMV and *rbohD/F*-TuMV interaction was characterized by upregulation of relative gene expression *AtGAUT1* and *AtGAUT7* accompanied by induction of highly methylesterificated HGs. Finally, an increase in cell wall methylesters level was revealed in both *rbohF*-TuMV and *rbohD/F*-TuMV interaction. It is indicated that induction of *GAUT1* and *GAUT7* gene expression, as seen in methylesterificated HGs synthesis [48], is in line with deposition of HG levels in both interactions, in contrast to susceptible *rbohD*-TuMV and Col-0-TuMV. Moreover, it was also reported that there is a close correlation between areas of deposition of GAUT1 and highly methylesterificated HGs (through JIM7) on the ultrastructural level during both resistance reactions to TuMV. However, it was postulated by Li et al. [115] that highly methylesterificated HGs were deposited at a 6 h timepoint during the interaction between resistance bananas on Foc4 (*Fusarium oxysporum* f., sp. cubense). On the other hand, Simon et al. [104] reported highly methylesterificated HGs recognized by JIM7 were induced during leaf pathogen *Cymadathea trifollii* infection. Furthermore, the significantly most intense localization of highly methylesterificated HGs was noticed in *rbohD/F*–TuMV leaf tissues accompanied by GAUT1, and was observed in the Golgi network, plasma membrane, and vesicular, membranous structures closely around and in the cell wall. However, the deposition of highly methylesterificated HGs together with GAUT1 indicated active distributions of HGs in resistance reactions to TuMV. HG is usually synthesized in the Golgi network [72,116] and can then be secreted into the cell wall in a highly methylesterificated form [117]. Engagement of plasma membrane and vesicular structures in GAUT and highly methylesterificated HG localization may confirm active distribution. Therefore, it can be assumed that during active cell wall rebuilding, AtPMEI2 and AtPMEI3 participated in *rbohF* and especially *rbohD/F* resistance reactions to TuMV, and it can be the effect of PME reduction activity. Moreover, reduced PME activity in that reaction resulted in a high content of cell wall methylesters and highly methylesterificated HGs. Induction of highly methylesterificated HGs seems to contribute to resistance reactions to TuMV—it modulates reduction in virus content or even virus transport.

Generally, many PMEI members can suggest the presence of dedicated, direct pairs of the specific PMEI–PME interactors, which can be an important factor in modulating demethylesterification in HG pectins [118] that determine cell wall rebuilding. Additionally, this fact can indicate that different PMEIs, like PMEI2 and PMEI3 in *rboh*-TuMV interactions, can target different PMEs to induce TuMV-dependent cell wall changes. This is a promising finding that needs further biochemical confirmation of a direct interaction between PMEI2 and PMEI3 with/or without PME3 as a response to TuMV.

## 4. Materials and Methods

### 4.1. Plant Material, Virus Inoculation, and Molecular Verification of TuMV Content

Cell wall remodeling in pectin-associated elements was checked in *A. thaliana* (L.) Heynh wild-type (Col-0) plants and selected specific mutants: *A. thaliana rbohD*, *rbohF*, and *rbohD/F* [27,28,119]. The Col-0 and mutant plants were selected based on different reactions to TuMV infection presented previously by Otulak-Kozieł et al. [27] and Otulak-Kozieł et al. [28]. All homozygous mutant seeds were kindly provided by Miguel-Angel Torres Laboratory. The plants were sown and cultivated according to the procedure and conditions presented by Otulak-Kozieł et al. [27,28]. All plants were next mock or TuMV inoculated, as described by Otulak-Kozieł et al. [120], Tomilson [121], and Walsh and Jenner [122]. For virus inoculation, we used TuMV inoculum (isolate PV-0104 was kindly provided by Leibniz Institute, Braunschweig, Germany) in phosphate buffer, as was presented [28,123]. The Col-0 and mutant plant leaves after 3, 7, and 21 dpi (days post-inoculation) of mock-inoculation and TuMV-inoculation were checked for the presence of the virus using qPCR (quantitative polymerase chain reaction). The verification of TuMV was based on the expression of the *TuMV-CP* gene in comparison to the mean expression of the plant host reference genes, *AtEf1α* and *AtF-Box*, as presented by Otulak-Kozieł et al. [28] and Otulak-Kozieł et al. [120], with use primers presented by Arous et al. [124] for *TuMV-CP*. The number of plants selected for all analyses was the same as presented by Otulak-Kozieł et al. [28] and performed in triplicate using a new set of plants every time. The mock-inoculated plants were free of TuMV. The results of the verification of the TuMV content are in Figure S1.

### 4.2. Analysis of Relative Expression of Selected AtPME, AtPMEI, and AtGSTU Genes in TuMV-Infected Col-0, rbohD, rbohF, and rbohD/F Plants Using qPCR

During the investigation of changes in pectin-associated elements associated with cell wall modification during TuMV infection, we performed the validation of the expression of selected genes. For analyses, we selected *Arabidopsis* genes: *AtPME3*, *AtPME17*, *AtPMEI2*, *AtPMEI3*, *AtGAUT1*, and *AtGAUT7*. The selection of genes for analyses was made based on two criteria: direct involvement of these genes in plant–pathogen interactions or a crucial role in pectin formation during cell wall building. Direct involvement in plant pathogen was reported in *AtPME3*, *AtPME17*, *AtPMEI2*, and *AtPMEI3*, respectively, by Raiola et al. [43], Del Corpo et al. [44], Lionetti et al. [45], Lionetti et al. [46], and Lionetti et al. [47]. For the core role in the synthesis of pectin homogalacturonan, we selected *AtGAUT1* and *AtGAUT* because they work together, as presented by Atmodjo et al. [48]. For the estimation of gene expression, we performed molecular analyses. Firstly, we collected leaf samples (0.1 g of each sample) at 3, 7, and 21 dpi from mock and TuMV-inoculated plants [120,125]. Then, according to the procedure presented in [120,125], we performed RNA isolation, purification, and quality checks, as well as confirmation of lack of RNA contamination. The absence of RNA contamination was also verified again by performing reverse transcription PCR using *AtEf1α* and *AtF-Box* as reference standards [28,125], which confirmed the absence of contaminating gDNA. Then, cDNA was synthesized using the NG dART RT Kit (EURx Sp. z o.o., Gdansk, Poland), as was presented by Otulak-Kozieł et al. [28]. Reverse transcription reactions were performed as described by Otulak-Kozieł et al. [125]. The number of analyzed plants was selected as presented by Otulak-Kozieł et al. [28].

Real-time qPCR for analyzed genes was performed according to procedure and with the use of the equipment described by Otulak-Kozieł et al. [125] and Otulak-Kozieł et al. [28] for *AtEf1α* and *AtF-Box* as reference genes. All qPCR tests were calibrated using previously prepared 6-point calibration curves (based on cDNA and gDNA). The analyzed *AtPME3*, *AtPME17*, *AtPMEI2*, *AtPMEI3*, *AtGAUT1*, and *AtGAUT7* were analyzed by qPCR in comparison to reference genes. The expression of these genes in *A. thaliana* was checked, and complete sequences were acquired from the TAIR database [126]. Based on previously published papers, the primers were chosen only for *AtPME17* [44]; the rest of the primers were designed using Primer3 software (version 0.4.0; Primer3Plus, Free Software Foundation, Inc., Boston, MA, USA). All the primers used during analyses are presented in Table S1. The starting cDNA solution (used for generating calibration curves) was prepared as presented by Otulak-Kozieł et al. [28]. An eightfold-diluted cDNA mix was used to construct the calibration curve for gDNA, while the subsequent calibration points were measured at four-fold dilutions in a 15 µL volume. A 5 µL solution of eight-fold-diluted cDNA mix was added to the reaction mixture. The exact conditions for qPCR analyses are shown in Table S2. The expression was analyzed statistically at selected time intervals for all plants by ANOVA.

### 4.3. Transmission Electron Microscope (TEM) Ultrastructural Analyses of Mock and TuMV-Inoculated Col-0, rbohD, rbohF, and rbohD/F Leaves

Fragments of leaves from mock/TuMV-inoculated Col-0, *rbohD*, rbohF, and *rbohD/F* plants at 3, 7, and 21 dpi were fixed and treated according to the procedure presented in [24,120,127] for TEM. Leaf samples were collected from a total of 60 plants (30 virus-inoculated and 30 mock-inoculated Col-0 and mutant plants of different types). The procedure was repeated three times, every time from a new group of plants. After TEM material preparation, the 50–70 nm sections from leaves prepared according to [24,127] were mounted on single slot copper grids from Agar Scientific (Stansted, United Kingdom: 0.75 mm Slot TEM copper Support Grids, catalog number: AGG2525C) coated with Formvar. The grids with samples were examined using transmission electron microscopy (TEM, FEI M268D “Morgagni” transmission electron microscope), as presented in [24,25], to check and characterize ultrastructural changes in the leaf cell wall. The observation in TEM was performed with a beam energy of 80 keV and snap exposure of 1.5 with the use of a SIS Morada digital camera (Olympus-SIS), and iTEM software version 5.0 (Olympus-SIS, Münster, Germany) to capture the images.

### 4.4. Bioinformatics Prediction of Subcellular Localization of PME3, PMEI2, PEMI3, and GAUT1 Proteins Based on Combined Predictor Database SUBA5 Search

The proteins selected for immunogold analyses were analyzed bioinformatically based on the online predictor SUBA5 [128] program (Table S3). SUBA5, as a group of online tools, enables the prediction of subcellular localization of proteins from *A. thaliana* based on different bioinformatic predictors. It also connects most protein predictor localization systems and new data from a mass spectrometry assay (MS/MS) gathered from the PubMed publication database [129] and integrates it into a suggested localization, the SUBA consensus (SUBAcon) [128,130]. The SUBAcon parameter only suggests a major/dominant place for localization but is coanalyzed with other predictors because most proteins are mobile in plant cells and could be also localized during in-cell transportation. For this complex prediction, we selected proteins whose genes significantly changed in reaction to the TuMV infection.

### 4.5. Quantified Immunogold Localization of PME3, PMEI2, PMEI3, GAUT1, and Two Types of HGs in Mock and TuMV-Inoculated Col-0, rbohD, rbohF, and rbohD/F Leaves

Samples prepared according to the TEM procedure as presented in Section 4.3 were also used for the preparation of 50–70 nm sections from leaves of all analyzed plants for immunogold localization. This section was next mounted to single-slot nickel grids from Agar Scientific (Stansted, United Kingdom: 0.75mm Slot TEM nickel Support Grids, catalog number: AGG2525N) coated with Formvar and treated according to the immunogold labeling procedure presented previously [24,125]. The immunogold labeling was performed to separately detect proteins PME3, PMEI2, PMEI3, and GAUT1, and two types of low/unesterificated HG (homogalacturonan) and highly methylesterificated HG. The selection of localized elements was made based on the results of gene expression and the bioinformatic analyses of the prediction of subcellular localization in SUBA5. For the detection of PME3, PMEI2, PMEI3, GAUT1, two types of low/unesterificated HG (homogalacturonan), and highly methylesterificated HG, we used different types of primary polyclonal/monoclonal antibodies targeting the selected elements. For the detection of PME3 (NCBI and TAIR protein numbers: NP_188048.1, AT3G14310.1), PMEI2 (NCBI and TAIR protein numbers: NP_188348.1, AT3G17220.1), and PMEI3 (TAIR protein numbers: AT5G20740.2), we used three custom-designed GeneCust (Boynes, France) polyclonal rabbit antibodies. Antibodies were designed separately for highly immunogenic C-terminal parts of PME3 (QGSGVKADATVAADGSGTFK), PMEI2 (GPSTCEQDMADFKVDPSA), and PMEI3 (NQLDETRGKPHDVHL). Sequences for designing were downloaded from NCBI and TAIR databases [126,131]. For the detection of GAUT1, we used rabbit polyclonal antibodies manufactured by PhytoAB Inc. (San Jose, CA, USA, catalog number: PHY1047S). In the case of low/unesterificated HG and highly methylesterificated HG, we used monoclonal rat antibodies JIM5 and JIM7, respectively, from the laboratory of Paul Knox, Ph.D., University of Leeds, United Kingdom, currently distributed by Agrisera (Vänäs, Sweden, catalog numbers: AS18 4194-1ml AS18 4195-1ml). The primary antibodies for immunogold localization were used in a 1:50 dilution in the case of the polyclonal rabbit antibodies (PME3, PMEI2, and PEMI3) and a 1:10 dilution in the case of the monoclonal rat antibodies (JIM5 and JIM7). Visualization was performed using secondary antirabbit or antirat antibodies conjugated with 18 nm nanogold particles (Jackson ImmunoResearch Europe Ltd., Cambridgeshire, UK, catalog numbers: 711-215-152, 112-215-143). The immunogold-labeled sections on the nickel grids were examined using a transmission electron microscope [24,26] with the parameters described in Section 4.3. Then, the localization of selected proteins and different types of HG was quantified following the method of Luschin-Ebengreuth and Zechmann [132] globally in the case of mock-inoculated and TuMV-inoculated plants. Statistical analyses were performed as described by Otulak-Kozieł et al. [24]. The concentrations of gold particles globally were validated using ANOVA and post hoc Tukey’s HSD (honest significant difference) test using Statistica software (version 13.0; StatSoft and TIBCO Software Inc., Palo Alto, CA, USA). The statistical estimation (with use of ANOVA) of immunogold labeling was performed for virus-inoculated and mock-inoculated samples and compared at 7 and 21 dpi (PME3, PMEI2, PMEI3, and GAUT1) and 21 dpi (low/unesterificated HG and highly methylesterificated HG). The number of gold particles globally was counted in 35 fields (10 μm^2^) per image. For each combination (mock-inoculated plants and TuMV-inoculated Col-0, *rbohD*, *rbohF*, and *rbohD/F* plants), gold particles from 200 photographs were counted to determine the presence of different proteins or HG.

### 4.6. Validation of PME Activity in Leaves of Mock and TuMV-Infected Col-0, rbohD, rbohF, and rbohD/F Plants

To validate the activity of PME, soluble protein extracts were generated from mock- and virus-inoculated leaves from Col-0, *rbohD*, *rbohF*, and *rbohD/F* plants from 7 and 21 dpi. For each combination, three replicates of extracts were generated. The leaf tissue from mock-inoculated and virus-inoculated plants was ground in liquid nitrogen and added to twice the fresh weight (*w*/*v*) of extraction buffer (100 mM Tris-HCl, pH 7.5, 500 mM NaCl) containing a protease inhibitor cocktail (ABMGood, Richmond, BC, Canada, catalog number: G135), as was described by Müller et al. [133] and Grsic-Rausch and Rausch [134]. Extracts from leaves were then rotated at 4 °C for 30 min and centrifuged at 11,500× *g* at 4 °C for 20 min. Fresh supernatants were used immediately for all enzyme assays, as presented by Müller et al. [133]. A coupled enzymatic assay was performed as described by Grsic-Rausch and Rausch (2004) using a spectrophotometric plate reader. For this purpose, we used Agilent BioTech Epoch 121221F, (Agilent, Santa Clara, CA, USA). The plate reader records the changes in absorption at 340 nm over 15 min at room temperature. The change in absorption per unit time over the linear part of the reaction was calculated for each well and used to calculate the increase in concentration of NADH. The NADH concentration was calculated using the extinction coefficient Ɛ_340_ for NADH (6220 M^−1^cm^−1^). PME activity was defined as U, where 1 U is 1 nmol of converted NADPH/s/mg total protein. The results were analyzed statistically using ANOVA and post hoc Tukey’s HSD (honest significant difference) test using Statistica software (version 13.0; StatSoft and TIBCO Software Inc., Palo Alto, CA, USA).

### 4.7. Validation of Cell Wall Methylesters in Mock and TuMV-Infected Col-0, rbohD, rbohF, and rbohD/F Leaves

To validate methylesters in the cell wall from mock- and virus-inoculated leaves of Col-0, *rbohD*, *rbohF*, and *rbohD/F* were then ground in liquid nitrogen, and 200 mL of methanol was added, as was previously described by Müller et al. [135]. The ground leaf tissue was extracted four times with a 1:1 (*v*/*v*) methanol:chloroform mixture, washed once with acetone, and dried overnight at room temperature. The weight of the dried cell wall materials was determined, and 0.5–1.0 mg was washed with 2 mL of water. To release the methylesters from the cell wall, the material was incubated for 1 h at room temperature with 100 mL of 0.5 M NaOH. After neutralization with 50 mL of 1 M HCl, the samples were centrifuged at 2000× *g* for 10 min. A generated supernatant was used to quantify the methanol, which was released during saponification according to the procedure presented by Klavons and Bennett [136] and Müller et al. [135] to generate extract from mock- and virus-inoculated leaves of Col-0, *rbohD*, *rbohF*, and *rbohD/F*. In the final step, the absorbance was measured at 412 nm using an Agilent BioTech Epoch 121221F (Agilent, USA) and compared with a standard curve generated with a methanol dilution series, as was presented by Müller et al. [135]. The results were analyzed statistically with the use of ANOVA and post hoc Tukey’s HSD (honest significant difference) test using Statistica software (version 13.0; StatSoft and TIBCO Software Inc., Palo Alto, CA, USA). Estimated methylester content was based on the methanol associated with saponified cell wall materials in leaves. Therefore, the estimated methylester content was presented as nmol methanol/mg cell wall material, as was suggested by Müller et al. [135].

## 5. Conclusions

These studies highlighted complex and dynamic cell wall modifications concentrated on selected elements/molecules associated with pectin metabolism during NADPH oxidase homologs D and F deficient *A. thaliana* mutants’ reaction to TuMV. Our findings indicated the importance of selected PME and PMEIs in regulating cell wall changes in Arabidopsis *rboh*-TuMV infections. The *rbohF*-TuMV and especially *rbohD/F*-TuMV mutant interactions displayed virus content limitation and dynamic cell wall rebuilding in all *A. thaliana* leaf tissues along with induced PMB formation or phenolic-like compound deposition, especially in vascular bundles. In contrast, Col-0 and *rbohD* mutants promoted TuMV infection and characterized cell wall rearrangement with induction of multivesicular structures and thickening in the plasmodesmata area in mesophyll and vascular tissue. As we confirmed previously [27], RbohF can promote increased susceptibility against TuMV. These susceptible reactions were displayed with upregulation of *AtPME3*, in contrast to *AtPME17*, which was also confirmed by induction of PME3 deposition. Our results revealed the highest PME activity in *rbohD*-TuMV, as well as a decrease in cell wall methylesters compared to mock-inoculated plants in both resistance interactions. Consequently, ultrastructural validation with quantification indicated that the susceptible reaction of *rbohD* and Col-0 to TuMV was characterized by significant domination of low/non-methylesterificated HGs. Conversely, cell wall rebuilding in the resistance response of *rbohF* and *rbohD/F* to TuMV was associated with dynamic induction of *AtPMEI2* and *AtPMEI3*, and also *AtGAUT1* with *AtGAUT7*, which was additionally confirmed by significant induction of the deposition of PMEI2, PMEI3, GAUT1, and GAUT7 proteins. Therefore, PMEI2 and PMEI3 can be important signaling resistance factors in the *rboh*-TuMV pathosystem. Cell wall changes in both resistance reactions were an effect of an intense decrease of PME activity, which was the most intense in *rbohD/F*-TuMV. It was accompanied by the induction of cell wall methylester content. Consequently, in contrast to susceptible reaction, dominated highly methylesterificated HGs were actively distributed while participating in *rbohF* and *rbohD/F* defense response and cell wall rebuilding. These results may help to provide new information on better understanding the mechanisms of defense response to TuMV. Further studies are needed to elucidate the confirmation of other components of cell wall structures participating in apoplast rearrangements in NADPH oxidase homologs D- and F-deficient mutants—the TuMV pathosystem.

## Data Availability

Data are contained within the article and Supplementary Materials.

## Supplementary Materials

The following supporting information can be downloaded at https://www.mdpi.com/article/10.3390/ijms25105256/s1.
