# Supplementary material for: Homogalacturonan Pectins Tuned as an Effect of Susceptible rbohD, Col-0-Reactions, and Resistance rbohF-, rbohD/F-Reactions to TuMV
Source: Int J Mol Sci. 2024 May 11;25(10):5256. doi: 10.3390/ijms25105256 (PMC11120978; doi:10.3390/ijms25105256)
Supplement: Supplementary file 1 [file ijms-25-05256-s001.zip › ijms-2970819-supplementary.pdf]

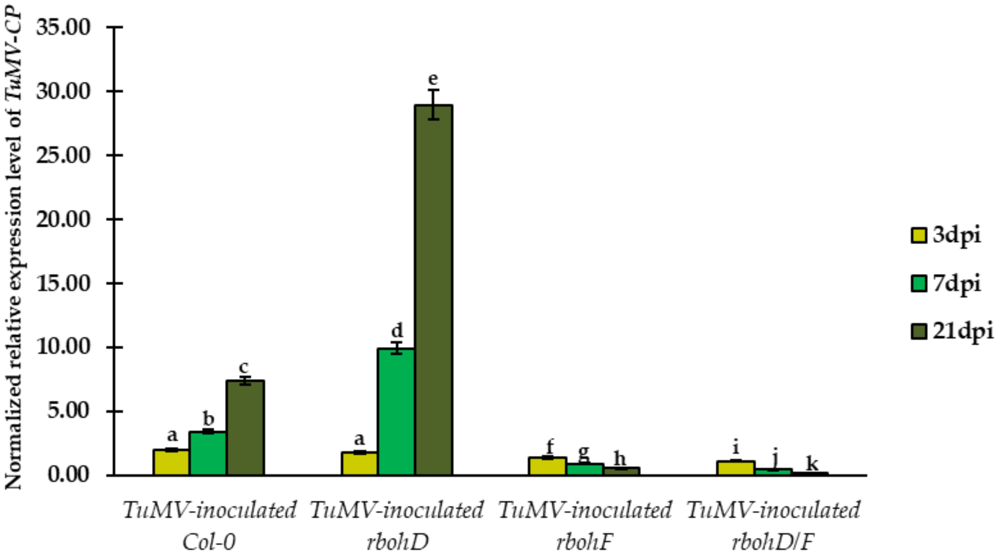

Figure S1. Validation of TuMV levels in Col-0, rbohD, rbohF, and rbohD/F plants at 3, 7, and 21 dpi based on normalized relative expression of *TuMV-CP*. Normalized relative expression of TuMV-CP was calculated based on the mean expression of *AtEf1α* and *AtF-Box* reference genes. Statistical significance of differences was assessed at the $p < 0.05$ level using ANOVA with post hoc Tukey's HSD and is indicated by different letters above the bars.

Table S1. Primer sequences for RT-qPCR analyses.

Genes	Forward Primer	Reverse Primer	Concentration in Reaction (μM)
		Invastigated	
TuMV-CP	5′-CCGGAATTCATGRTITGGTGYATIGAIAAYGG-3′	5′-CGCGGATCCGCIGYYTTCATYTGIRIIWKIGC-3′	0.5
AtPME3	5′-CTCCGCCATTACCAACCA -3′	5′- CGTGTATCTGCCCCTTCAA -3	0.5
AtPME17	5′-GAGAGGTGATGGTATAGGGA -3′	5′- GAGCTGTGATTGTGTTGGTC -3′	0.5
AtPMEI2	5′-CTAGTCAAGACAGCAACCAA -3′	5′- AGGCTCTGCTTAGCATCA -3′	0.5
AtPMEI3	5′-TCCGTACTCTTTCGTCATACTC -3′	5′- CTGATTTTTATGGCGGCTTGA -3′	0,5
AtGAUT1	5′-AGTTTCCACAGATTCGACAAG -3′	5′- CCATAAGCCCATCCACAAG -3′	0.5
AtGAUT7	5′-GGTTTGAATGTCGTTGATCTTG -3′	5′- ACTCATCTCCACTACTCATCTC -3′	0.5

Genes	Forward Primer	Reverse Primer	Concentration in Reaction (μM)
		Reference	
AtEF1a	5′-CACCACTGGAGGTTTTGAGG -3′	3′- TGGAGTATTTGGGGGTGGT -5′	0.5
AtF-Box	5′-GCTTGCACACGCCATATCAAT-3′	3′-TGGATTTTACCACCTTCCGCA-5′	0.5

Table S2. Conditions of the RT-qPCR for the reference genes (*).

Program	Parameters
Preliminary denaturation	95 °C for 10 min
Amplification (40 cycles)	95 °C for 15 s<br>58-61°C for 30 s<br>72 °C for 30 s *
Melting curve	65–95 °C; 0.1 °C/s

* Fluorescence signal reading was taken at the final stage

Table S3. Predictions of protein localization according different predictors connected with SUBA5 prediction and integrated in database PubMed MS/MS results

Protein name	Results of bionforamtic subcellular localization predictions with predictors allocated in SUBA5	SUBAcon	MS/MS data
**AtPME3**	Golgi apparatus (predictors: PredSL, PProwler)<br>Plastid (predictors: EpiLoc, PCLR)<br>Vacuole (predictor: YLoc)<br>Mitochondrion (predictors: iPSORT, SLPFA and SubLoc)<br>Extracellular/cell wall (predictors: PredSL, PProwler, Plant-mPloc, TargetP)<br>Nucleus (predictors: BaCelLo, SLP-Local)<br>endoplasmic reticulum (predictors: TargetP, PredSL, PProwler)<br>cytosol (Predictors: AdaBoost)<br>plasma membrane (predictors: WoLF PSORT)	Extracellular/cell wall	Extracellular/cell wall,<br>Golgi apparatus/Trans Golgi Network,<br>Multivesicular body<br>Mitochondrion,<br>Nucleus,<br>Plasma membrane<br>Plastid,<br>Vacuole,
**AtPMEI2**	Golgi apparatus (predictors: Predotar, PredSL, PProwler, SLPFA, SLP-Local, TargetP)<br>Plastid (predictors: PCLR, WoLF PSORT, ChloroP)<br>Mitochondrion (predictors: iPSORT, Mitoprot 2, SubLoc)<br>Extracellular/ cell wall (predictors: Predotar, PredSL, PProwler, SLPFA, SLP-Local, TargetP, YLoc, MultiLoc)<br>Nucleus (predictors: BaCelLo)<br>Endoplasmic reticulum (predictors: TargetP, Predotar, PredSL, PProwler, SLPFA, SLP-Local)<br>Plasma membrane (predictors: AdaBoost, Plant-mPloc)	Extracellular/cell wall	Nucleus
**AtPMEI3**	Golgi apparatus (predictors: Predotar, PredSL, PProwler, iPSORT, SLP-Local, TargetP)<br>Plastid (predictors: AdaBoost, WoLF PSORT)<br>Nucleus (predictors: BaCelLo)<br>Extracellular/ cell wall (predictors: iPSORT, SLP-Local, TargetP, YLoc, Predotar, PredSL, PProwler)<br>Endoplasmic reticulum (predictors: TargetP, Predotar, PredSL, PProwler, iPSORT, MultiLoc, SLP-Local)<br>Plasma membrane (predictors: Plant-mPloc)<br>Mitochondrion (predictors: SLPFA, SubLoc)	Extracellular/cell wall	Extracellular/cell wall
**AtGAUT1**	Golgi apparatus (predictors: EpiLoc, MultiLoc, YLoc)<br>Plastid (predictors: Plant-mPloc, BaCelLo)<br>Mitochondrion (predictors: MitoPred, PredSL, SLPFA, SubLoc, iPSORT, Predotar, PProwler, SLP-Local, TargetP)<br>Cytosol (predictors: WoLF PSORT)<br>Plasma membrane (predictors: AdaBoost)	Golgi apparatus	Extracellular/cell wall,<br>Golgi apparatus/Trans Golgi Network<br>Multivesicular body<br>Plasma membrane<br>Plastid<br>Vacuole
